# Catheter ablation in patients with ventricular fibrillation by purkinje de-networking

**DOI:** 10.3389/fcvm.2022.956627

**Published:** 2022-09-30

**Authors:** Vanessa Sciacca, Thomas Fink, Denise Guckel, Mustapha El Hamriti, Moneeb Khalaph, Martin Braun, Christian Sohns, Philipp Sommer, Guram Imnadze

**Affiliations:** Clinic for Electrophysiology, Herz- und Diabeteszentrum Nordrhein-Westfalen, Ruhr-Universität Bochum, Bad Oeynhausen, Germany

**Keywords:** Purkinje de-networking, Purkinje system, ventricular fibrillation, ventricular fibrillation ablation, sudden cardiac death

## Abstract

**Background:**

Ventricular fibrillation (VF) is a leading cause of cardiovascular death worldwide. However, recurrence rates of arrhythmia are high leading to mortality and morbidity. Recently, Purkinje fibers have been identified as potential sources of VF initiation and maintenance.

**Aim:**

The study analyzes the feasibility and effectiveness of catheter ablation in patients with recurrent VF by specific Purkinje de-networking (PDN).

**Methods:**

Consecutive patients with recurrent VF undergoing PDN were included in this observational study. The procedural endpoint was the non-inducibility of sustained ventricular arrhythmia. A three-dimensional -anatomical mapping was conducted, and the specific cardiac conduction system and Purkinje fibers were tagged. All detectable Purkinje signals were ablated in the left ventricle (LV). Additional right ventricular (RV) PDN was performed in case of VF inducibility after LV ablation. Follow-up was performed by patient visits at our outpatient clinic including device interrogation and by telephone interviews.

**Results:**

Eight patients were included in the study. Six patients were females (75%); the median age during the procedure was 43 [37;57] years and the median body mass index was 24 [23;33] kg/m^2^. Four patients (50%) had known structural heart disease with two cases of ischemic cardiomyopathy and two cases of dilated cardiomyopathy. In four patients (50%), no underlying structural heart disease could be identified. The median LV ejection fraction was 42 ± 16.4%. All patients had an implantable cardioverter-defibrillator (ICD) prior to ablation with documentation of recurrent VF. The median number of ICD shocks before the ablation was 5 [3;7]. LV PDN was performed in all patients. In two patients (25%), an additional RV PDN was performed. Non-inducibility of any ventricular arrhythmia was achieved in all patients after PDN. Two patients showed complete left bundle branch block post-ablation. The median follow-up duration was 264 [58;421] days. Two patients (25%) experienced ventricular arrhythmia recurrence with recurrent ICD-shock delivery. One patient died during follow-up with an unknown cause of death. Six patients (75%) experienced no arrhythmia recurrence during follow-up.

**Conclusion:**

Purkinje de-networking represents a novel treatment option for patients with recurrent VF without arrhythmia substrate or specific arrhythmia triggers with promising results in terms of efficiency and feasibility. Larger and more prospective studies are needed for a systematic evaluation.

## Introduction

Sudden cardiac death is a leading cause of cardiovascular mortality worldwide with 50–85% being attributable to ventricular fibrillation (VF) (1, 2). However, the etiology in VF is heterogeneous. Structural heart disease, such as coronary artery disease or other structural cardiomyopathies are common findings, especially in elderly patients with VF (3). Nevertheless, VF also occurs in patients with apparently structural normal hearts. Distinct electrical phenotypes, such as Brugada syndrome or Long-QT syndrome have been identified as causes of VF in structurally normal hearts (4). However, VF remains unexplained in a considerable number of patients without evidence of structural or electrical myocardial disease. The term idiopathic VF has been introduced to describe this special entity of VF (5). Within the past two decades, relevant progress in the understanding of initiation and maintenance of VF in the context of structural heart disease as well as in terms of idiopathic VF has been made. Experimental studies were able to identify a wide spectrum of activation patterns, such as stable three-dimensional rotors or multiple wavelets and foci to be associated with VF (6–8). Interestingly, the Purkinje system, as a distinct anatomical region, has been shown to play an active role in triggering and initiating VF (9). Moreover, the Purkinje system was shown to be an essential component of sustaining VF (10). Implantable cardioverter-defibrillators (ICDs), as secondary prevention and antiarrhythmic drugs, are the mainstay of therapy in patients with VF, to date. However, recurrence rates of arrhythmia are high leading to mortality and morbidity. Additional treatment strategies are warranted to offer patients with VF better symptom control. Current guidelines recommend catheter ablation in patients with scar-related or ischemic heart disease with incessant ventricular tachycardia (VT) and electrical storm or with recurrent adequate ICD shocks (11). There are no general recommendations in terms of catheter ablation among patients without structural heart disease and without overt triggers or substrate but recurrent VF or fast polymorphic VTs are implemented in current guidelines. The growing body of evidence on the major role of the Purkinje network in generating automatic and triggered focal rhythms as well as accommodating re-entrant circuits support the fact that the Purkinje network promotes initiation and maintenance of VF and substrate ablation of VF by targeting Purkinje fibers which may represent a promising novel treatment option for patients with recurrent VF in the absence of arrhythmogenic substrate or triggers. Systematic targeting of the Purkinje system during catheter ablation has been proposed before and has been referred to as “Purkinje de-networking” (PDN) (12). The present study analyzes the feasibility and effectiveness of catheter ablation by PDN in patients with recurrent VF or fast polymorphic VT for reduction of arrhythmia burden.

## Materials and methods

### Study population

Data from consecutive patients who underwent radiofrequency-based catheter ablation for recurrent VF or fast polymorphic VT between December 2019 and February 2022, at our institution, were analyzed. Exclusion criteria consisting of documented monomorphic VT documented the trigger of VT or VF in the form of distinct ventricular premature beats and documented VF as the final event of monomorphic VT, ventricular thrombus, recent myocardial ischemia (within 6 months prior to ablation), or patients’ unwillingness to participate. All patients gave written informed consent before participating. The study followed the principles outlined in the Declaration of Helsinki and was approved by the local ethics committee (2019-563). The study was conducted as a prospective observational study and as a part of our institutional catheter ablation registry. Baseline, procedural, and follow-up data were collected and statistically analyzed.

### Peri- and intraprocedural management

All patients underwent transthoracic echocardiography before ablation to rule -out the possibility of ventricular thrombi. In patients who are at high risk of left atrial or left atrial appendage thrombi, transesophageal echocardiography was performed prior to ablation. Interrogation of cardiac devices was performed before ablation, and in cases of ICD, deactivation of tachycardia therapy was conducted. Patients with medication of vitamin-k antagonists received uninterrupted anticoagulation with a target international normalized ratio (INR) from 2.0 to 3.0. In patients with chronic use of direct oral anticoagulants (DOACs), uptake was paused for one half-life before the procedure and continued within 6 h after ablation. All procedures were performed in deep analgetic sedation based on continuous propofol infusion and fractionated administration of midazolam and fentanyl. During ablation, continuous monitoring of heart rate, oxygen pressure, invasive measurement of blood pressure, and body temperature was performed. Heparin administration for systemic anticoagulation was started after venous access targeting an activated clotting time (ACT) that is greater than 300 s.

### Ablation protocol

**FIGURE 1:**
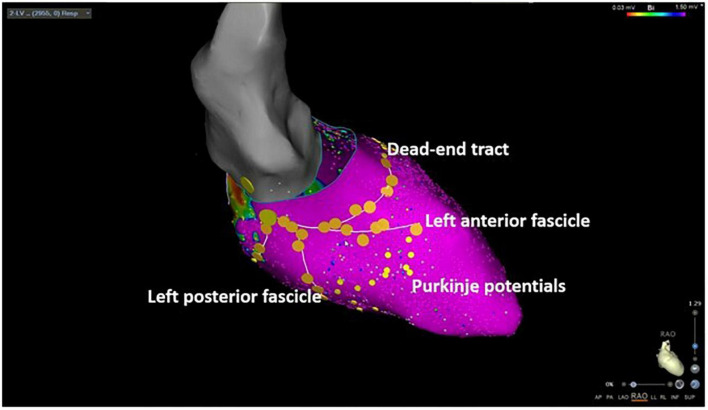
Three-dimensional electroanatomic mapping of a patient undergoing selective Purkinje de-networking (PDN) due to recurrent ventricular fibrillation. The left anterior and left posterior fascicles as well as the dead-end tract were marked by large yellow tag points. Purkinje potentials were marked by small yellow tag points. The target region for ablation was the area between the left anterior and left posterior fascicle aiming at the elimination of all visualized Purkinje potentials.

**FIGURE 2:**
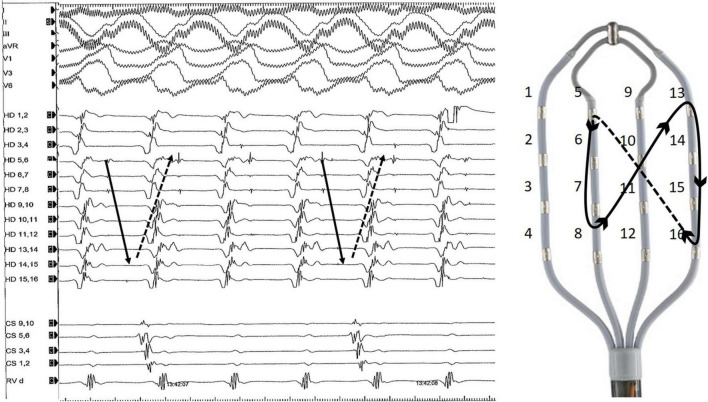
Representative example of diastolic Purkinje potentials during an organized ventricular arrhythmia. The tachycardia was induced by programmed right ventricular stimulation and degenerated into VF. On the right side of the figure is an example of a multipolar diagnostic catheter (HD Grid, Abbott) and Purkinje activation trajectory.

The detailed ablation approach was published before (12). After venous femoral access, diagnostic catheters were placed in the right ventricle (RV) and coronary sinus. *Trans*-septal puncture for left heart access was then performed under fluoroscopic guidance with an SL1-sheath (St. Jude, Abbott, Abbott Park, IL, USA) which was replaced by a steerable long sheath (Agilis, Long Curl, Abbott). After left ventricular (LV) access, a 3D electroanatomic mapping was performed using a mapping system (Carto 3, Biosense Webster, Irvine, CA, USA or Ensite Precision, Abbott). Multipolar mapping catheters were used for LV electroanatomic mapping (Pentaray, Biosense Webster, or Advisor HD Grid Mapping Catheter, Abbott). An anatomical and bipolar voltage map of the LV was created in the sinus rhythm with a setting of 0.5–1.5 mV to distinguish the normal tissue from the abnormal tissue and to detect endocardial scarring. The left conduction system with the left main branch, left anterior, and left posterior fascicle l as well as Purkinje potentials along the left-sided conduction system including the dead-end tract and the lateral aspect of the papillary muscle were marked as colored tag points (Figure 1). RV mapping was performed analogously alongside the right bundle. All Purkinje potentials were tagged with the use of multipolar mapping catheters and were confirmed by mapping with the ablation catheter during the procedures. After LV and RV mapping and tachycardia induction was performed by programed RV stimulation with up to four extra beats. The multipolar mapping catheter was positioned before arrhythmia induction at the LV septum. In the case of tachycardia induction, activation mapping registration of Purkinje potentials was performed for the longest possible time in terms of hemodynamic stability (Figure 2). Radiofrequency ablation was performed by replacing the mapping catheter with an irrigated tip bidirectional D/F curve ablation catheter (SmartTouch^®^, Biosense Webster or FlexAbility™, Abbott, USA). Ablation was performed over 30–40 s with 30–35 Watt of power. Radiofrequency ablation was performed between the left anterior and left posterior fascicle by PDN in the sense of tissue homogenization. Purkinje spike elimination was then performed alongside the dead-end tract and at papillary muscle sites. At the end of the procedure, RV programed stimulation with up to four extra stimuli was performed aiming at tachycardia induction. RV ablation was performed analogously according to individual detectable Purkinje signals in case of inducibility of VF or VT after LV ablation. Ablation at the septal side of the moderator band was confirmed by 3D electroanatomic mapping and transthoracic echocardiography.

### Postprocedural management

Pericardial effusion was ruled out by transthoracic echocardiography immediately after the procedure and before the patient left the electrophysiology laboratory. Vessel access sites were closed with a figure-of-eight suture. After device interrogation and reactivation of tachycardia therapy, all patients were transferred to a wake-up area. After an observational period of 2–4 h, patients were transferred to the general ward. The groin suture was removed the day after ablation. During the hospital stay, heart rhythm was continuously monitored by telemetry. The regularly scheduled in-hospital stay after ablation was 2 days.

### Follow-up

Clinical follow-up (FU) was conducted at our outpatient clinic after 3, 6, and 12 months including an assessment of the clinical history and 12-lead-ECG. Device interrogation was conducted at presentation and by remote monitoring every 4 weeks or immediately in case of recurrence of arrhythmia. Subsequently, clinical visits were conducted every 12 months at our outpatient clinic. Arrhythmia recurrence was defined as any documented sustained ventricular arrhythmia with or without ICD therapy. Inadequate ICD therapy due to atrial arrhythmia or sensing issues was not counted as ventricular arrhythmia recurrence after ablation.

### Statistical analysis

Continuous data were summarized as the median [1st; 3rd quartile] and categorical data as absolute and relative frequencies (n, %). All *p*-values were two-sided and a *p*-value < 0.05 was considered statistically significant. All calculations were performed with statistical analysis software (IBM^®^ SPSS^®^).

## Results

### Baseline characteristics

Eight patients with recurrent VF or polymorphic VT undergoing PDN were included in the study. Six patients (75%) were females. The median age at the timepoint of ablation was 43 [37;57] years. The median body mass index was 24 [23;33] kg/m^2^. Median LV ejection fraction was 49 [38;53]%. Coronary angiography was performed in all patients prior to ablation in the context of diagnostic work-up of underlying heart disease. Furthermore, cardiac MRI was performed on all patients during the initial diagnostic workup. None of the patients showed abnormalities that were specific to a certain type of cardiomyopathy and no specific areas of distinct scarring could be detected at the time of MRI. Structural heart disease was present in four (50%) patients. Of these, two patients (50%) suffered from ischemic cardiomyopathy due to coronary artery disease and the other two from dilative cardiomyopathy. In four patients (50%) no underlying structural or primary electrical disease was diagnosed despite intensive workup. No patient had a family history of sudden cardiac death. All patients were ICD-carriers prior to ablation. Six of the eight patients (75%) had received ICD for secondary prevention after survival of sudden cardiac death. Two patients (25%) received an ICD for primary prevention. One of the patients in this collection had a cardiac resynchronization system. Median ventricular pacing rates in patients without a cardiac resynchronization system were low at 17% [8;23]. The clinical arrhythmia was VF in four patients (50%) and fast polymorphic VT in the other four patients (50%). All patients had received adequate ICD shock delivery for VF or VT before undergoing ablation with a median number of ICD shocks of 5 [3;7]. All patients experienced ICD shock delivery before ablation despite antiarrhythmic drug therapy with a class I or class III medication. Oral anticoagulation with DOAC was present in two patients (25%) and with vitamin-K-antagonists in one patient (12.5%). Multiple antiarrhythmic drug therapy was administered in all patients before catheter ablation. Stellate ganglion blockade was not performed prior to ablation in this patient cohort. Prior ventricular ablation was also not performed in any of the patients. Detailed information on baseline demographics is presented in Table 1.

**TABLE 1:** Baseline characteristics.

Male, n (%)	2 (25)
Female, n (%)	6 (75)
Age (years)	43 [37;57]
BMI (kg/m^2^)	24 [23;33]
Coronary angiography, n (%)	8 (100)
Structural heart disease, n (%)	4 (50)
Coronary artery disease, n (%)	2 (25)
Dilative cardiomyopathy, n (%)	2 (25)
LV-EF (%)	49 [38;53]
Family history of sudden cardiac death, n (%)	0 (0)
ECG trigger documented, n (%)	0 (0)
ICD carrier, n (%)	8 (100)
VF as clinical arrhythmia, n (%)	4 (50)
Fast polymorphic VT as clinical arrhythmia, n (%)	4 (50)
ICD shock delivery before ablation, n (%)	8 (100)
Number of ICD shocks before ablation	5 [3;7]
Antiarrhythmic drug before ablation, n (%)	8 (100)
Oral anticoagulation, n (%)	3 (37.5)

BMI, body mass index; LV-EF, left ventricular ejection fraction; ECG, electrocardiogram; ICD, implantable cardioverter-defibrillator; VF, ventricular fibrillation; VT, ventricular tachycardia.

### Procedural characteristics

The median procedural duration was 162 [141;175] min. The median fluoroscopy time was 10 [8;12] min and the median radiation dosage was 275 [160;416] cGy*cm^2^. Electroanatomic mapping could not reveal relevant arrhythmogenic substrates in all patients. LV ablation was performed in all patients. In two patients, RV ablation was performed due to persistent inducibility of VF after left-sided PDN. None of the patients in this cohort experienced major procedure-related complications. Details on procedural characteristics are presented in Table 2.

**TABLE 2:** Procedural characteristics.

Procedure duration (min)	162 [141;175]
Fluoroscopy time (min)	10 [8;12]
Radiation dosage (cGy*cm^2^)	275 [160;416]
Contrast (ml)	10 [7;15]
Additional substrate	0 (0)
Ablation site LV, n (%)	6 (75)
Ablation site LV and RV, n (%)	2 (25)
Inducibility at the end of procedure, n (%)	0 (0)
Complete left bundle branch block after PDN, n (%)	2 (25)

LV, left ventricle; RV, right ventricle; PDN, Purkinje de-networking.

### Intraprocedural findings

**FIGURE 3:**
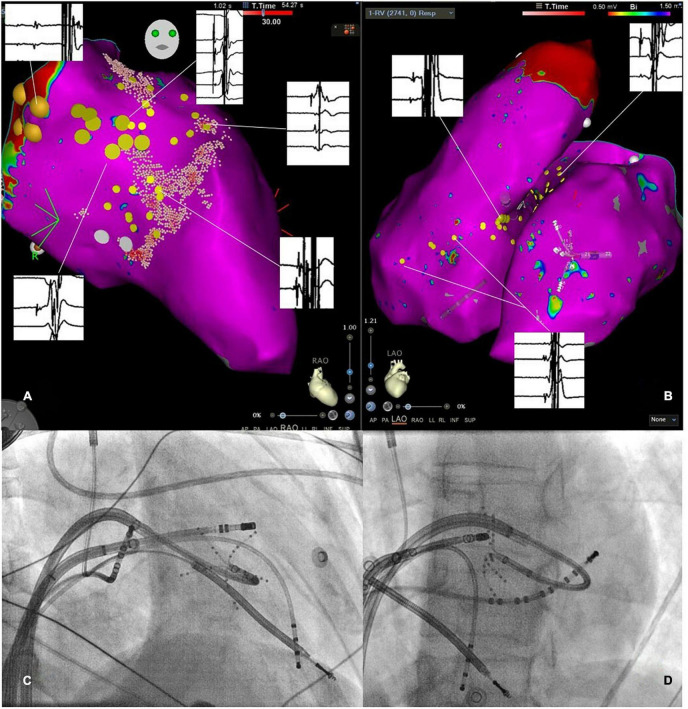
**(A)** Three-dimensional electroanatomic mapping of the left ventricle (LV) in right anterior oblique projection. The specific conduction system has been marked by large yellow tag points representing the His-bundle region and the left anterior fascicle. Regions with distinct Purkinje potentials were marked with small yellow tag points. Representative electrocardiograms are shown and linked to the specific location of the recording. Notably, clear Purkinje potentials are observed. **(B)** Three-dimensional electroanatomic map in the left anterior oblique view of the RV and LV with small yellow tag points placed at regions with Purkinje potentials. Exemplary electrograms are linked to the specific site of a recording showing clear Purkinje potentials. **(C,D)** Show fluoroscopic catheter-set up in the right anterior oblique and left anterior oblique view consisting of a multipolar mapping catheter at the LV septum, two diagnostic catheters placed in the RV and the coronary sinus as well as an ablation catheter in the RV.

During LV and RV mapping, none of the patients in this cohort showed relevant arrhythmic substrate. Further ablation of scar areas and late potentials was therefore not performed. Other potential triggers of ventricular arrhythmias, such as spontaneous premature ventricular contractions, were not observed in this patient cohort and therefore were not targeted during ablation. Notably, left bundle branch potentials and at least 3–4 distal Purkinje potentials could be mapped in every patient. In three patients (37.5%), fast polymorphic VT or VF was easily inducible by catheter movement or programed stimulation. In these patients, focal activation patterns at mid-apical septal regions during tachycardia, as well as mid-diastolic Purkinje potentials could be observed. In the other five patients (62.5%), mechanical or programed arrhythmia induction was not possible. However, distinctive Purkinje potentials were observed in all of these patients. Figure 3 shows a representative three-dimensional electroanatomic mapping of one of the patients without relevant low voltage areas below 1.5 mV and with distinct Purkinje potentials. PDN was performed in all patients by substrate modification of the region between the left anterior and left posterior fascicle as well as the dead-end tract. In two patients (25%), right bundle branch de-networking at the septal site of the moderator band was performed due to the persistent inducibility of clinical ventricular arrhythmia. At the end of the procedure, non-inducibility of any ventricular arrhythmia could be achieved in all patients. A novel complete left bundle branch block was observed in two patients (25%) at the end of the procedure.

### Follow-up results

**FIGURE 4:**
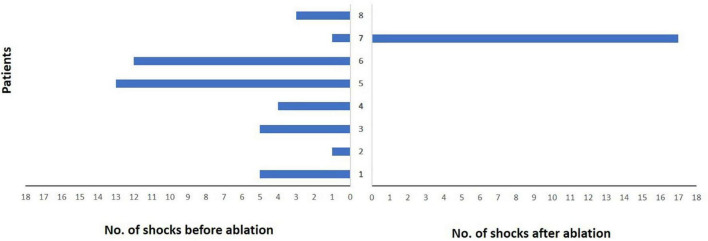
Burden of implantable cardioverter-defibrillator (ICD) shock delivery per patient during follow-up after ablation. Each patient is represented by a single bar in the diagram. Only one patient did not experience ICD shock reduction due to recurrent ventricular tachycardia storm. However, a significant reduction in the median number of ICD shocks could be observed in the other patients.

The median follow-up duration was 264 [58;421] days. Two patients (25%) experienced recurrent ventricular arrhythmia with VF after 45 days in one case, and polymorphic VT after 85 days in the other case. The patient with recurrent VF experienced a VF storm with 17 ICD shocks and underwent implantation of a LV assist device as well as repeat endocardial ablation at another center. The patient with recurrent polymorphic VT did not experience symptoms during arrhythmia recurrence, and device interrogation revealed successful termination after two cycles of ATP. Despite one patient having a recurrent VF storm, the ICD shock burden could be effectively reduced in seven patients (87.5%) (Figure 4). Median time to ventricular arrhythmia recurrence after ablation was 65 [55;75] days. One patient (12.5%) died 6 months after ablation with an unknown cause of death. Two patients (25%) were on antiarrhythmic drug therapy with either a class I or class III medication at the timepoint of the last follow-up. All patients continued beta-blocker medication. Two patients experienced a new complete left bundle branch block after PDN. One patient had a cardiac resynchronization device before ablation and did not experience any clinical deterioration. The other patient with a new complete left bundle branch block was a carrier of a transvenous one lead ICD and had an LV ejection fraction of 52%. No deterioration of LV ejection fraction or symptoms of heart failure occurred during the follow-up period of this patient. Hence, an upgrade to cardiac resynchronization therapy was not indicated. Details on follow-up results are presented in Table 3.

**TABLE 3:** Follow-up results.

Follow up duration (days)	264 [58;421]
VF recurrence, n (%)	1 (12.5)
Polymorphic VT recurrence, n (%)	1 (12.5)
Time to recurrence (days)	65 [55;75]
Patients with shock delivery post-ablation, n (%)	1 (12.5)
Patients with ATP delivery post-ablation, n (%)	1 (12.5)
Repeat ablation, n (%)	1 (12.5)
Antiarrhythmic drug at last follow-up, n (%)	3 (37.5)
Death during follow-up, n (%)	1 (12.5)

VF, ventricular fibrillation; VT, ventricular tachycardia; ATP, anti tachycardia pacing.

## Discussion

The present study analyzed the feasibility and effectiveness of catheter ablation in patients with recurrent VF or fast polymorphic VT in the absence of regional endocardial substrate or distinct triggers by specific de-networking of the Purkinje system. The main findings of the present study are as follows:

1. Catheter ablation by PDN in patients with recurrent VF or polymorphic VT in the absence of regional substrate or distinct triggers was effective in terms of reducing arrhythmia recurrence and ICD shock burden.
2. Catheter ablation by PDN was technically feasible with acceptable procedure durations and low complication rates.
3. Biventricular ablation to achieve non-inducibility of VF or polymorphic VTs was necessary for about 25% of patients.

### Mechanisms of initiation and maintenance of ventricular fibrillation and other polymorphic ventricular arrhythmias

Monomorphic and stable ventricular arrhythmias occur typically in the presence of myocardial scarring, most notably in patients with previous myocardial infarction. Polymorphic ventricular arrhythmias have several causes, including myocardial ischemia and electrolyte disturbances leading typically to polymorphic VT or VF. Nevertheless, the mechanisms of VF are only poorly understood. The role of the Purkinje system in the initiation of VF and other ventricular arrhythmias was hypothesized already years ago. Leenhardt et al. found short-coupled premature ventricular complexes initiating Torsade de points tachycardia with frequent degeneration into VF (13). These arrhythmias were only amendable by verapamil therapy, suggesting the role of the His-Purkinje system in arrhythmia initiation. Years later, successful catheter ablation of patients with electrical storm shortly after myocardial infarction targeting premature ventricular complexes arising from the Purkinje system was reported (14). It was hypothesized that degeneration of Purkinje fibers in border zones of myocardial infarction areas led to malignant arrhythmias. Catheter ablation of premature ventricular contractions originating in these regions was effective in arrhythmia suppression in multiple studies (15). In a study by Nakamura et al., patients with idiopathic VF underwent ablation of frequent premature ventricular contractions as potential VF triggers. During follow-up, 77% of patients with VF remained free from arrhythmia recurrence (16). A recent study by Haissaguerre et al. investigated 54 patients with VF in the presence of structural or electrical heart disease (10). The authors used non-invasive and invasive mapping methods and found a stable electrical activation of VF during a short initiation phase, which was amenable for mapping. Patients underwent ablation of early ventricular or Purkinje activation. Of note, surrounding ablation was performed in case of early Purkinje activation during ablation. In a case report by Hasegawa et al., frequent episodes of VF were mapped under extracorporeal membrane oxygenation and a nearly regular Purkinje activation could be demonstrated during long-lasting arrhythmia episodes (17). Ablation resulted in the termination of VF within minutes after the ablation of Purkinje signals in this case. While the patient did not survive due to hemodynamic instability and cerebral hypoxemia, the case highlights the potential role of Purkinje fibers not only in initiation but also in the maintenance of VF.

### Purkinje de-networking

Previous studies on VF ablation found VF triggers in form of frequent premature ventricular contractions, which were consequently targeted for catheter ablation (14–16). Nevertheless, VF without the identification of clear trigger mechanisms is a frequent clinical problem, and mapping via specialized tools is usually not available in a routine clinical setting. No drug therapy except amiodarone has proven reliable effectiveness in VF suppression to date. Since many patients with VF are of younger age, long-time antiarrhythmic therapy often cannot be used as a long-term therapy due to toxic side effects and quality of life issues. Consequently, there is a clear need for novel effective treatment options. PDN may represent an effective treatment in a selected patient cohort without other therapeutic options. Mapping of the conduction system and Purkinje potentials is reproducible and relatively easy. We performed PDN in our case series and found it to be feasible, safe, and effective for arrhythmia suppression. PDN as an anatomical ablation approach is not dependent on arrhythmia inducibility and arrhythmia mapping, which might be challenging or impossible due to hemodynamic instability. In our cohort, arrhythmia recurrence was observed in two patients after PDN, which seems comparable to results obtained by Haissaguerre et al. (10). In the above-mentioned studies by Haissaguerre and Nakamura, 61 and 77% of patients were free of ICD therapies during follow-up, which is also in line with our results. PDN might also represent an additional ablation maneuver in patients with fast VT or VF and electroanatomic substrate. However, further studies on larger patient cohorts with longer follow-up durations are needed to confirm our initial results.

### Limitations

The study has the typical limitations of an observational study regarding data quality. Furthermore, the study population is small and there was no control group. The patients analyzed in this cohort were heterogeneous in terms of an underlying disease which limits the generalizability of the presented results. Larger and prospective studies are needed to further validate the potential benefits of PDN in patients with recurrent VF and without specific triggers or relevant substrates.

## Conclusion

Catheter ablation in patients with recurrent VF or fast polymorphic VT without specific endocardial substrate or overt arrhythmia triggered by PDN was feasible and effective in terms of reducing arrhythmia recurrence and ICD shocks. Larger trials are needed to further validate the concept of PDN as a novel treatment option in this special patient cohort.

## Data availability statement

The original contributions presented in this study are included in the article/supplementary material, further inquiries can be directed to the corresponding author.

## Ethics statement

The studies involving human participants were reviewed and approved by the Ethics Committee of the Ruhr-Universität Bochum Health Campus, Bochum. The patients/participants provided their written informed consent to participate in this study.

## Author contributions

VS, PS, and GI: concept of the manuscript. VS and GI: methodology and figures. VS: data acquisition, data analysis, and manuscript preparation. TF, DG, ME, MK, MB, CS, PS, and GI: manuscript revision. TF, MK, CS, DG, and PS: validation. VS, TF, and PS: editing and reviewing. PS: supervision. All authors contributed to the article and approved the submitted version.
